# Chromatin remodeling modulates radiosensitivity of the daughter cells derived from cell population exposed to low- and high-LET irradiation

**DOI:** 10.18632/oncotarget.17275

**Published:** 2017-04-20

**Authors:** Ping Wang, Dexiao Yuan, Fei Guo, Xiaoyan Chen, Lin Zhu, Hang Zhang, Chen Wang, Chunlin Shao

**Affiliations:** ^1^ Institute of Radiation Medicine, Fudan University, Shanghai 200032, China

**Keywords:** radiosensitivity, LET-dependent, daughter cells, chromatin remodeling, heterochromatinization

## Abstract

Radiation effects are dependent of linear energy transfer (LET), but it is still obscure whether the daughter cells (DCs) derived from irradiated population are radioresistance and much less the underlying mechanism. With the measurements of survival, proliferation and γH2AX foci, this study shows that the DCs from γ-ray irradiated cells (DCs-γ) became more radioresistant than its parent control without irradiation, but the radiosensitivity of DCs from α-particle irradiated cells (DCs-α) was not altered. After irradiation with equivalent doses of γ-rays and α-particles, the foci number of histone H3 lysine 9 dimethylation (H3K9me3) and the activity of histone deacetylase (HDAC) in DCs-γ was extensively higher than these in DCs-α and its parent control, indicating that a higher level of heterochromatin was formed in DCs-γ but not in DCs-α. Treatment of cells with SAHA (an inhibitor of HDAC) decreased the level of heterochromatin domains by inhibiting the expressions of H3K9m3 and HP-1a proteins and triggering the expression of acetylated core histone H3 (Ac-H3). When cells were treated with SAHA, the radioresistance phenotype of DCs-γ was eliminated so that the radiosensitivities of DCs-γ, DCs-α and their parent cells approached to same levels. Our current results reveal that γ-rays but not α-particles could induce chromatin remodeling and heterochromatinization which results in the occurrence of radioresistance of DCs, indicating that the combination treatment of irradiation and HDAC inhibitor could serve as a potential cancer therapy strategy, especially for the fraction radiotherapy of low-LET irradiation.

## INTRODUCTION

Fraction radiotherapy is widely used for the treatment of malignant tumors since its advantages in the preservation of normal tissue damage. However, tumor radioresistance remains a major therapeutic problem to a successful cancer therapy [1]. It has been reported that tumor could effectively evade from radiotherapy by a number of strategies including its own defense system in selection of radioresistant cells so that the tumors become more and more radioresistant during fraction radiotherapy [2–4], which gives a clue that the daughter cells (DCs) derived from an irradiated cell population may have a lower radiosensitivity than its parent control.

Accumulating evidence suggests that the epigenetic mechanisms, such as histone modification and DNA methylation associated with abnormal gene expressions, contribute to the intrinsic radioresistance of cancer cells by promoting cell proliferation and DNA damage repair [5–7]. Histone acetylation is a major protein modification mode in relaxing chromatin structure and promoting gene transcription and it is controlled by histone acetyltransferases (HATs) and histone deacetylases (HDACs). HATs can acetylate lysine of histone protein, cause chromatin structure relaxation, and facilitate gene activation. Conversely, HDACs could catalyze the removal of acetyl group from lysine residue in histone protein, induce the condensation of chromatin structure, and inhibit gene expressions [8]. There are many lines of evidence that the compacted structure of chromatin can decrease radiation-induced damage in DNA. Study of poly-nucleosomes showed that radiation-induced DNA double strand breaks (DSBs) were not randomly distributed along DNA molecule but rather preferentially localized in the linker regions (euchromatin regions), while the core regions (heterochromatin regions) of DNA were more resistant to radiation [9, 10]. Isolated DNA of euchromatin was about 4-fold more susceptible to DSB than that of heterochromatin [9]. This differential radiosensitivity is apparently due to the close association of chromatin structure [9, 10]. Therefore cell radiosensitivity relies on the chromatin structure and dynamics.

Numerous studies have shown that HDACs can activate DNA-damage repair (DDR) responses because they are critical in modulating chromatin remodeling and maintaining dynamic acetylation equilibrium of DNA damage related proteins [11]. Interfering pharmacologically with HDAC activity can correct abnormalities in cell proliferation, migration, vascularization and death [12]. HDAC inhibitors (HDACi) could change the balance between the deacetylating activity of HDACs and the acetylating activity of HATs, which leads to increase of histone acetylation and up-regulation of its gene expression [8]. Enhancement of histone acetylation could result in modification of gene expressions in a variety of tumors [13]. Although the biological function of HDAC has been well studied, little is known about its activity alteration after radiation.

Recently, the clinical use of charged particle therapy has gained significant interests worldwide. High-LET radiation has greater potential to cause serious DNA damage because it has a higher relative biological effectiveness (RBE) and its efficacy of cytocidal action is approximately 3-fold greater than that of photons [14]. Our previous studies have shown that both long-term radiations of low-LET γ-rays and high-LET α-particles could induce radiation adaptive response and even enhanced the potential of malignant transformation of offspring cells [6, 15]. Although the chromatin remodeling induced by histone acetylation modifications can activate radiation response, the differences of radiosensitivity and chromatin structure remolding in the DCs derived from cell population exposed by different LET radiations are largely unclear.

In this study, we investigated the radiosensitivity of DCs from lung normal cells Beas-2B and cancer cells A549 exposed to different-LET radiations and then explored the relationship between cell cytocidal actions and chromatin remodeling in these different DCs. Results showed that a low-LET irradiation increased HDAC activity and induced heterochromatinization and radioresistance of DCs. Conversely, a high-LET irradiation rarely destined the DCs’ radiosensitivity and chromatin status. The underlying molecular mechanisms of these findings were further disclosed.

## RESULTS

### DCs of γ-ray irradiated cells were more resistant to irradiation than that of α-particle exposed cells

**Figure 1:**
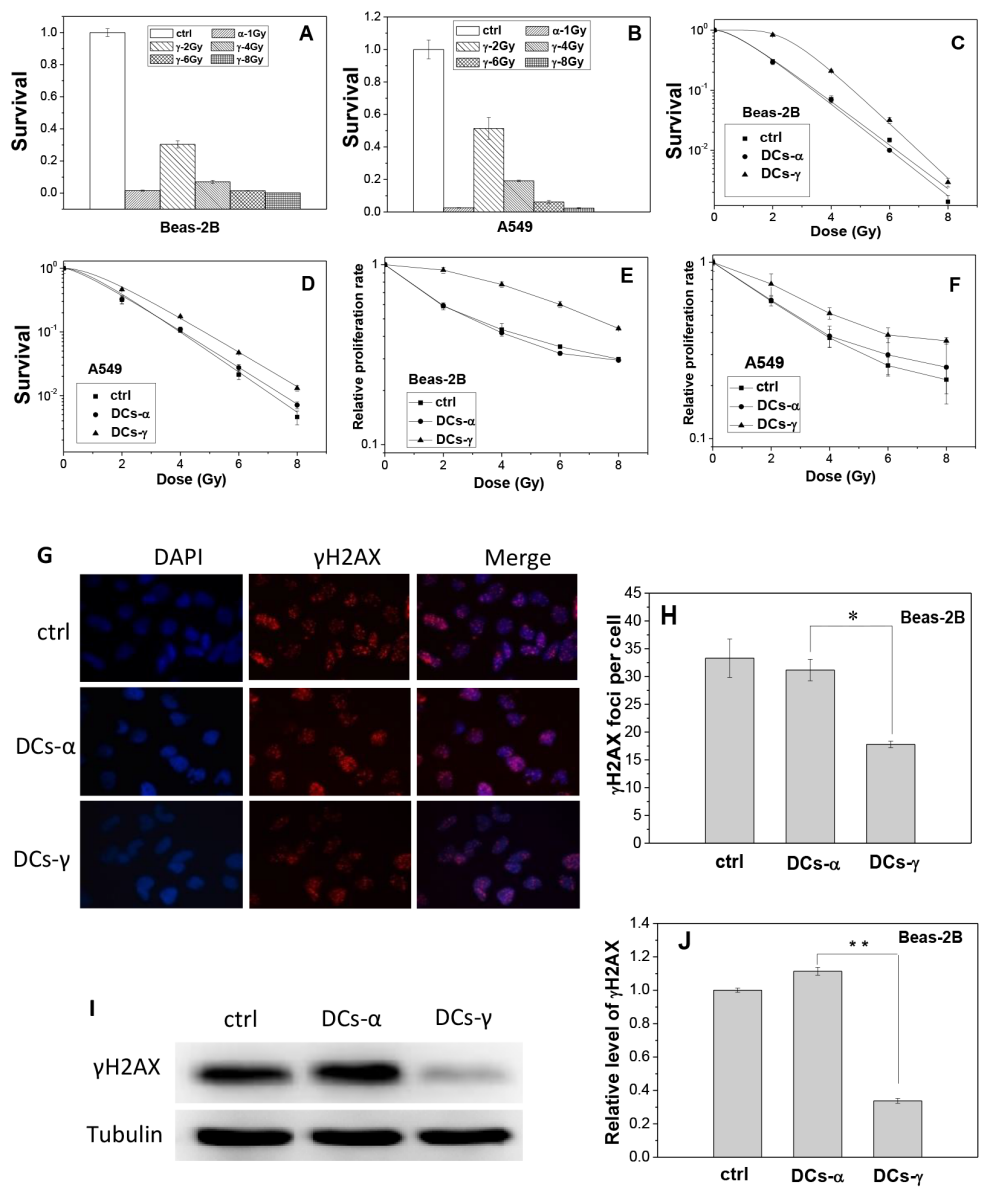
Survival fraction, proliferation and DNA damage of lung cells and their daughter cells (DCs) Beas-2B cells and A549 cells were irradiated with priming doses of γ-rays and α-particles, respectively. Then the irradiated cells were cultured for 2 weeks to obtain their DCs. **(A, B)** Clonogenic survivals of Beas-2B and A549 cells irradiated by 1 Gy α-particles or 2, 4, 6, and 8 Gy γ-rays. **(C, D)** Clonogenic survivals of DCs-α, DCs-γ and its parent control irradiated with different test doses of γ-rays. **(E, F)** Proliferation rate of DCs-α, DCs-γ and its parent control irradiated with different test doses of γ-rays. **(G, H)** γH2AX foci in the DCs-α and DCs-γ of Beas-2B cells. The DCs were irradiated with a test dose of 2 Gy γ-rays and fixed immediately after irradiation for immunostaining assay of γH2AX foci. The foci were counted in at least 200 cells. **(I, J)** Protein expressions of γH2AX in the DCs-α, DCs-γ and its parent control. Proteins were determined by Western blotting and normalized to its corresponding level of β-Tubulin. Data were presented as means ± SEMs of three independent experiments. * *P* < 0.05.

To properly evaluate the difference of radiosensitivity of DCs exposed to low- and high-LET radiations, the DCs were generated from the offspring of the cells irradiated with equivalent biological dose of γ-rays and α-particles, respectively. Clonogenic survival assay showed that 1 Gy of α-particles and 6 Gy of γ-rays had similar biological effect on normal lung cells Beas-2B (Figure 1A), while 1 Gy of α-particles and 8 Gy of γ-rays had similar biological effect on lung cancer cells A549 (Figure 1B). After 2-3 weeks of these priming irradiation, the surviving DCs of irradiated cells were collected for further mechanistic investigations.

It was found that, for both Beas-2B and A549 cells, when the DCs derived from γ-ray irradiated cells (DCs-γ) were further irradiated by γ-rays with test doses of 2, 4, 6 and 8 Gy, its clonogenic survival and cell proliferation rate were significantly higher than those of its parent control cells without priming irradiation (Figure 1C and 1E); but when the DCs derived from α-particle irradiated cells (DCs-α) were irradiated with these test doses, its clonogenic survival and cell proliferation rate were similar to those of its parent control cells (Figure 1D and 1F). Moreover, when the DCs of Beas-2B cells and its parent control were irradiated with 2 Gy γ-rays, the level of phosphorylated histone H2AX (γH2AX) in DCs-γ but not DCs-α was obviously lower than that in the control (Figure 1G and 1H). In consistent, after 2 Gy irradiation, the expression level of γH2AX protein in DCs-γ but not DCs-α were only 34% of that in its parent Beas-2B cells (Figure 1I and 1J). These results reveal that, in comparison with high-LET α-particle irradiation, the priming irradiation of low-LET γ-rays was more able to have DCs to be radioresistance.

### Higher level of heterochromatin was induced in DCs-γ rather than DCs-α

**Figure 2:**
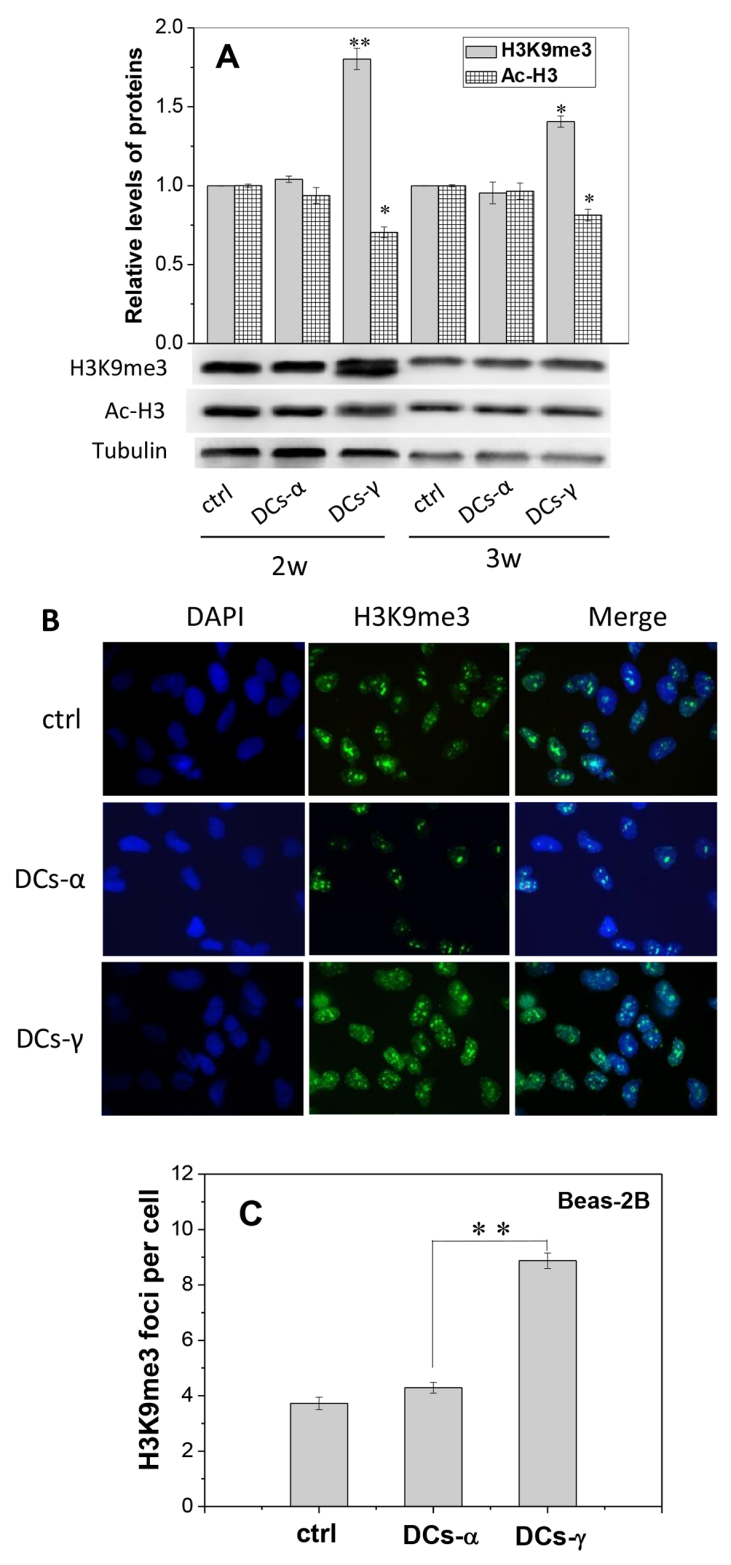
Expressions of H3K9me3 and Ac-H3 in the DCs of irradiated Beas-2B cells Beas-2B cells were irradiated with 1 Gy α-particles or 6 Gy γ-rays, respectively, and then cultured for 2-3 weeks to obtain DCs-α and DCs-γ. **(A)** Protein expressions of H3K9me3 and Ac-H3 in the DCs-α, DCs-γ and its parent control. Proteins were determined by Western blotting and normalized to its corresponding level of β-Tubulin. **(B, C)** Immunostaining images of H3K9me3 foci and its number in DCs-α, DCs-γ and their parent control cells. The foci were counted in at least 200 cells. Data were presented as means ± SEMs of three independent experiments. * *P* < 0.05, ** *P* < 0.01.

The different radiosensitivity of DCs-γ and DCs-α may result from the chromatin remodeling after priming irradiation. To testify this assumption, we measured the expressions of relevant proteins involved in chromatin structure in Beas-2B cells. Figure 2A showed that after 6 Gy of priming γ-ray exposure, the protein expression of H3K9me3, the marker of heterochromatin, in DCs-γ increased to 1.80-fold and 1.41-fold of control after two- and three-weeks of irradiation, respectively. The expression of acetylated core histone H3 (Ac-H3) in DCs-γ was reduced to about 70% of control cells after two weeks of priming γ-ray irradiation. However, after 2-3 weeks of priming α-particle irradiation, the expressions of both H3K9me3 and Ac-H3 in DCs-α had no significant changes in comparison with that in nonirradiated cells.

The foci of heterochromatin marker H3K9me3 in the nuclear of DCs were also measured after two-weeks of priming irradiation. As shown in the immunofluorescence staining images (Figure 2B), the number of H3K9me3 foci in DCs-γ was 2.07-fold of that in DCs-α (Figure 2C). These findings demonstrate that low-LET irradiation could induce chromatin remodeling by increasing heterochromatin domains, which may eventually lead to cell radioresistance.

### Enhancement of HDAC activity in DCs

**Figure 3:**
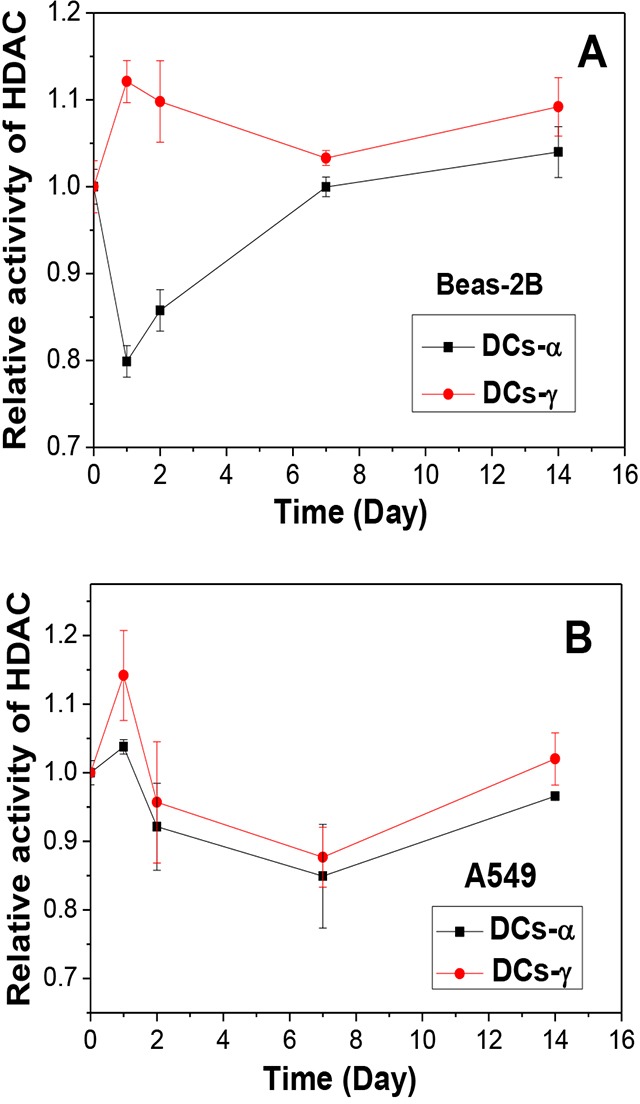
Time responses of HDAC activity in DCs-α and DCs-γ after priming irradiation **(A)** Beas-2B cells were irradiated with α-particles and γ-rays at equivalent priming doses of 1 Gy and 6 Gy, respectively. **(B)** A549 cells were irradiated with α-particles and γ-rays at equivalent priming doses of 1 Gy and 8 Gy, respectively. Data were presented as means ± SEMs of three independent experiments.

To know the reason of heterochromatinization occurred in DCs-γ but not in DCs-α, we investigated whether the activity of HDAC is different in DCs-γ and DCs-α. Figure 3A confirms that, after one day of priming irradiation, the HDAC activity was increased by 12% in DCs-γ but decreased by 20% in DCs-α of Beas-2B cells. With extension of cell culture time after irradiation, the HDAC activity in DCs-γ gradually decreased but it was still higher than that in DCs-α even two weeks after irradiation. For A549 cells, the HDAC activity in DCs-γ was also higher than that in DCs-α (Figure 3B). This observation elucidates that low-LET irradiation was much more effective in activating HDAC and promoting heterochromatinization than high-LET irradiation.

### Inhibiting HDAC activity caused chromosome decondensation and radioresistant

**Figure 4:**
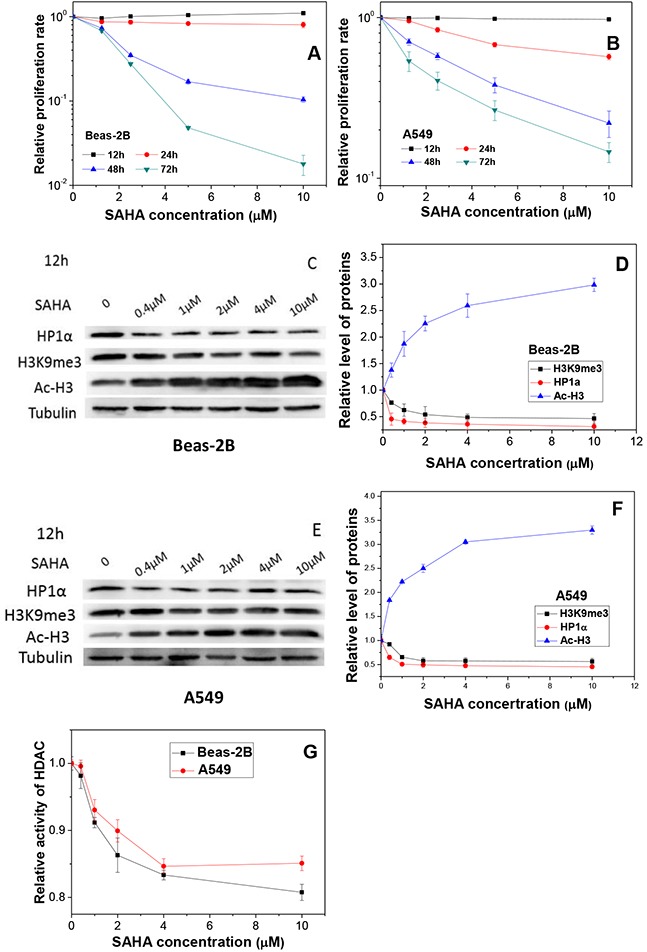
Influence of SAHA in cell proliferation, HDAC activity and chromatin status **(A, B)** The proliferation of Beas-2B and A549 cells treated with different concentrations of SAHA for different periods. **(C, E)** Protein expressions and their relative levels of HP1a, H3K9me3 and Ac-H3 in Beas-2B and A549 cells treated with different concentrations of SAHA for 12 h. **(D, F)** Proteins were detected by Western blotting and normalized to its corresponding level of β-Tubulin. **(G)** HDAC activities in Beas-2B and A549 cells treated with different concentrations of SAHA for 12 h. Data were presented as means ± SEMs of four independent experiments.

To further verify the role of chromosome structure in radiosensitivity of DCs, the cells were treated with SAHA, a HDAC inhibitor. To have a suitable treatment condition of the inhibitor, the toxic effect of SAHA was detected in Beas-2B and A549 cells. When Beas-2B and A549 cells were treated with SAHA of different concentrations from 0 to 10 μM for different time from 12 h to 72 h, it was found that the cell proliferation was not influenced by SAHA within 12 h of treatment, but the cell growth was inhibited by SAHA in a dose-dependent manner when the treatment time was longer than 24 h (Figure 4A and 4B). Accordingly, we treated cells with 2 μM SAHA for 12 h in further experiments.

This SAHA treatment increased the acetylation of histone H3 protein extensively but reduced the expressions of heterochromatin marker H3K9me3 and HP1α proteins in a dose-dependent manner in Beas-2B (Figure 4C and 4D) and A549 (Figure 4E and 4F), respectively. Figure 4G shows that the HDAC activity of both Beas-2B and A549 cells decreased rapidly with the increase of SAHA concentration.

**Figure 5:**
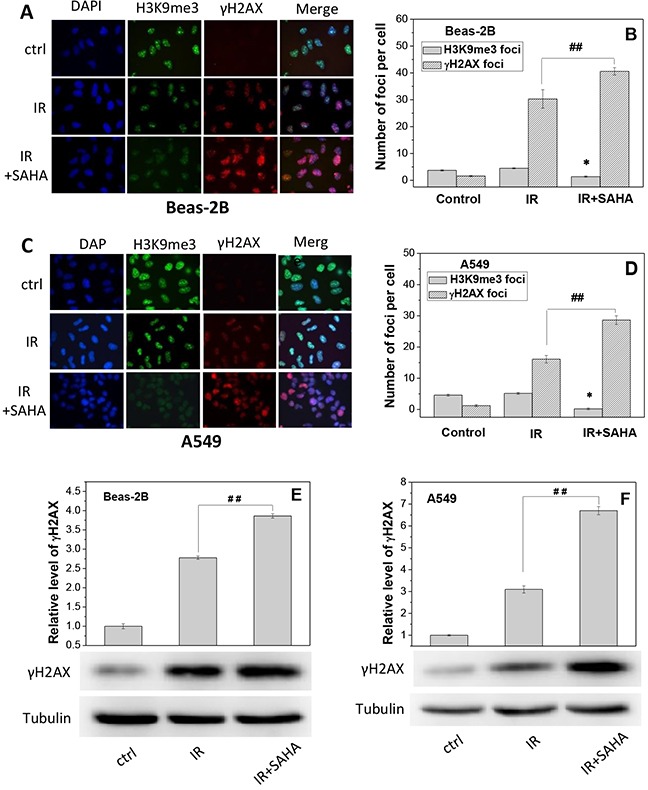
Treatment of cells with SAHA increases radiation damage by decondensating chromosome structure Beas-2B and A549 cells were irradiated with 2 Gy g-rays with or without treatment of 2 μM SAHA for 12 h. H3K9me3 and gH2AX foci were measured immediately after priming irradiation. **(A, C)** Immunostaining images of H3K9me3 and gH2AX foci in Beas-2B cells and A549 cells. **(B, D)** The foci numbers of H3K9me3 and γH2AX counted in at least 200 cells of Beas-2B and A549. Data were presented as means ± SEMs of three independent experiments. **(E, F)** Protein expressions of γH2AX in Beas-2B and A549 cells after SAHA treatment. Proteins were determined by Western blotting and normalized to its corresponding level of β-Tubulin. * *P* < 0.05 and ## *P* < 0.01 compared to corresponding irradiated cells.

To understand the influence of SAHA in chromatin structure and DNA damage, both Beas-2B and A549 cells were treated with SAHA for 12 h and then irradiated with 2 Gy γ-rays. It was found that, after irradiation, the number of H3K9me3 foci was rarely altered but the number of γH2AX foci was obviously increased for both Beas-2B and A549 cells (Figure 5). Moreover, treatment of cells with SAHA decreased the number of H3K9me3 foci by 70% and 95.5% but increased the number of γH2AX foci by 34% and 78% in the irradiated Beas-2B and A549 cells, respectively (Figure 5B and 5D). Western blotting assay of γH2AX protein also shows that, after SAHA treatment, the expression level of γH2AX increased to 1.4-fold and 2.2-fold of Beas-2B (Figure 5E) and A549 cells (Figure 5F) that merely irradiated with priming dose. These findings elucidate that radiation-induced DNA damage can be increased when the heterochromatin domains in cells were reduced by SAHA treatment.

### HDAC inhibitor eliminates radioresistance of DCs by decondensating chromosome structure

**Figure 6:**
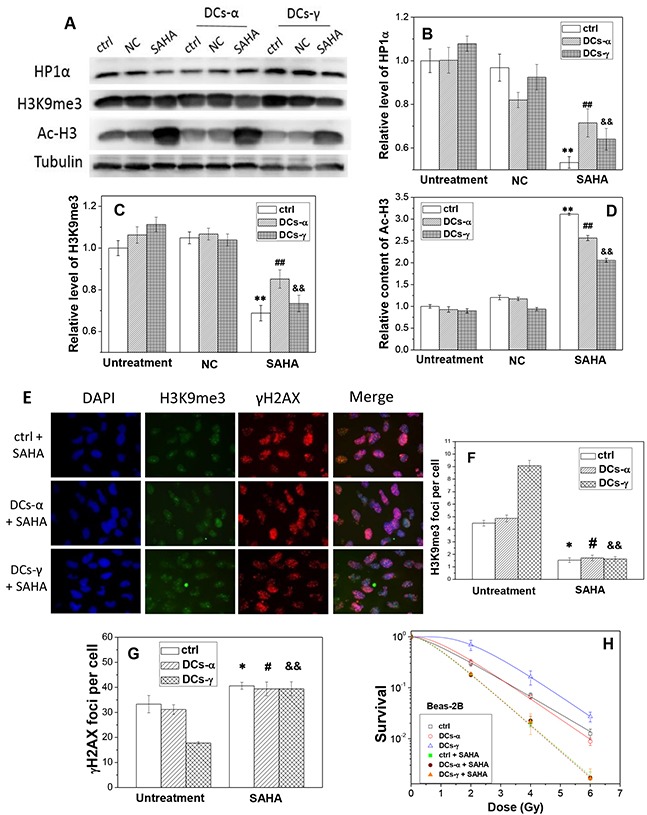
The treatment of cells with SAHA decreases the level of heterochromatin and increases radiosensitivities of DCs-α and DCs-γ of Beas-2B cells DCs were treated with 2 μM SAHA for 12h. **(A)** Western blotting image of HP1α, H3K9me3 and Ac-H3 in DCs and its parent control. Ctrl: control cells with irradiation; NC: negative control of SAHA. **(B, C, D)** Relative expression levels of HP1α, H3K9me3 and Ac-H3 in the DCs and its parent control cells with and without SAHA treatment. Tubulin was used as an internal reference. **, ## and &&, *P* < 0.01 compared to corresponding NC group. **(E)** Immunostaining images of H3K9me3 and gH2AX foci in DCs-α and DCs-γ. DCs were treated with 2 μM SAHA for 12 h and irradiated again with a test dose of 2Gy γ-rays, then fixed immediately after irradiation for immunostaining assay of H3K9me3 and γH2AX. Cell nuclear were stained with DAPI. **(F, G)** The foci number of H3K9me3 and γH2AX in both DCs and its parent control cells after test irradiation of 2 Gy γ-rays. Foci number were counted in at least 200 cells. * and #, *P* < 0.05 compared to corresponding control. &&, *P* < 0.01 compared to corresponding control. **(H)** Dose responses of the survival of DCs and its parent control with or without SAHA treatment. Cells were irradiated with different test doses of γ-rays. Data were presented as means ± SEMs of three independent experiments.

To further verify the relationship between HDAC activity and radiosensitivity, we measured the heterochromatin protein levels and radiosensitivity of DCs after treatment of cells with HDAC inhibitor. Western blotting assay shows that, after SAHA treatment, the expressions of the heterochromatin marker HP1α in both DCs and its parent cells of Beas-2B decreased by 15-45% (Figure 6A and 6B) and the levels of H3K9me3 reduced by 20-35% (Figure 6C). While in contrast the content of Ac-H3 was increased to about 2.2-3.2 fold of negative control (NC) (Figure 6D) in both DCs-γ and DCs-α and their parent cells.

**Figure 7:**
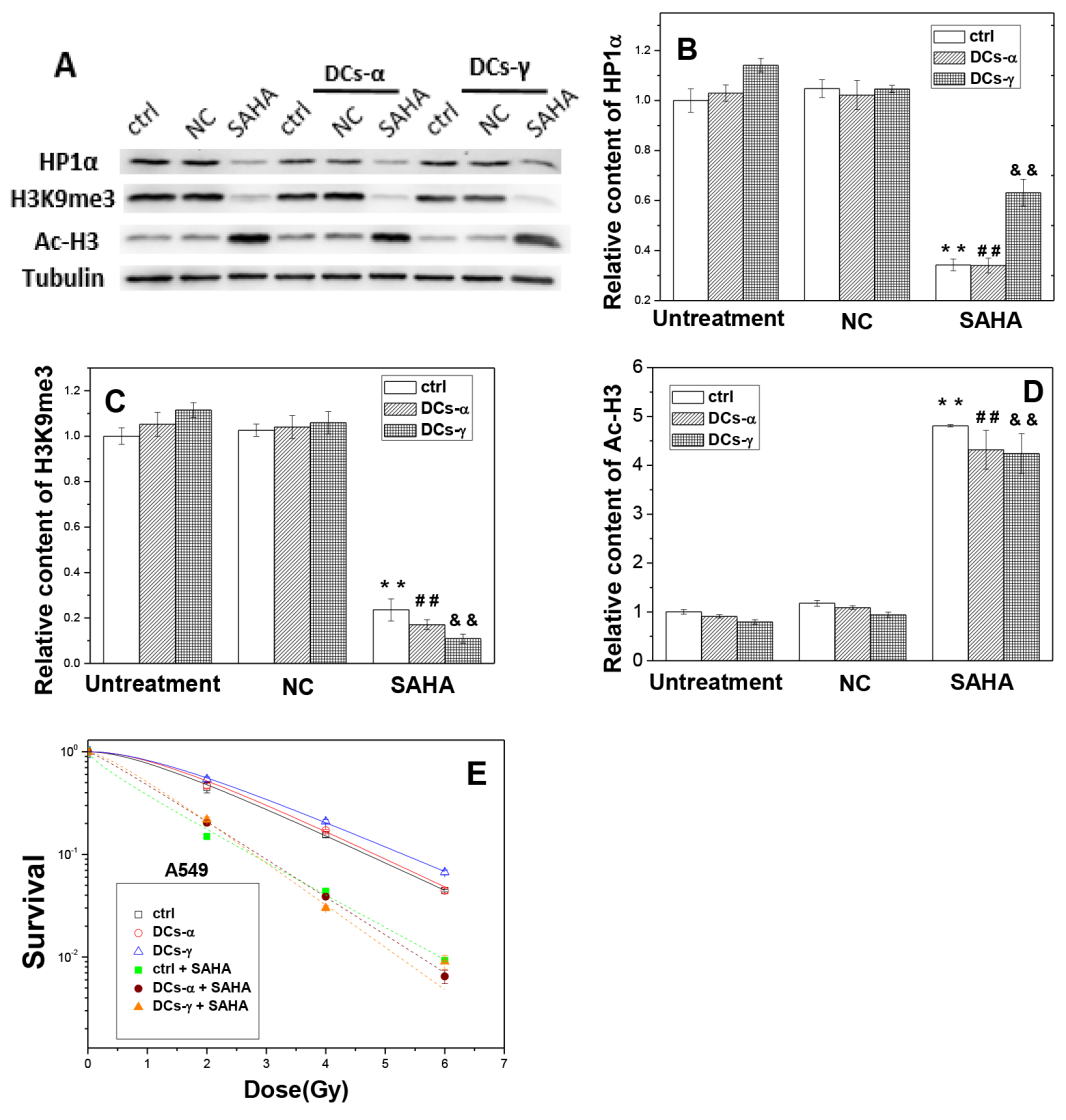
The treatment of cells with SAHA decreases the level of heterochromatin and increases radiosensitivities of DCs-α and DCs-γ of A549 cells DCs were treated with 2 μM SAHA for 12 h. **(A)** Western blotting image of HP1α, H3K9me3 and Ac-H3 in DCs and its parent control. Ctrl: control cells with irradiation; NC: negative control of SAHA. **(B, C, D)** Relative expression levels of HP1α, H3K9me3 and Ac-H3 in the DCs and its parent control cells with and without SAHA treatment. Tubulin was used as an internal reference. **, ## and &&, *P* < 0.01 compared to corresponding NC group. **(E)** Dose responses of the survival of DCs and its parent control with or without SAHA treatment. Cells were irradiated with different test doses of γ-rays. Data were presented as means ± SEMs of three independent experiments.

Similar results were found in A549 cells and its DCs. After SAHA treatment, the expressions of HP1α decreased by 40-67% (Figure 7A and 7B) and the levels of H3K9me3 reduced by 77-90% (Figure 7C), while the content of Ac-H3 was increased to about 4-fold of negative control (NC) (Figure 7D) in both DCs-γ and DCs-α and their control without priming irradiation. As a result, the HDAC inhibitor diminished radiation induced heterochromatin in accompany with extensive increase of acetylation of histones.

To determine whether HDAC activity has a direct effect on cell radiosensitivity, we evaluated the influence of SAHA treatment on the induction of γH2AX foci and the clonogenic survival of DCs. When DCs and its parent Beas-2B cells without priming irradiation were treated with SAHA for 12 h and then irradiated with a test dose of 2 Gy γ-rays, the number of H3K9me3 foci decreased and the number of γH2AX foci increased dramatically (Figure 6E). For the DCs-γ, the SAHA treatment reduced H3K9me3 foci number by 82% and increased γH2AX foci number by 2.2-times (Figure 6F and 6G). Interestingly, for the DCs-α, the treatment of HDAC inhibitor allowed the levels of H3K9me3 and γH2AX to be similar to those in DCs-γ. In addition, the clonogenic survival measurement shows that, this SAHA treatment increased the radiosensitivities of both DCs-γ and DCs-α and their parent control without priming irradiation to a same level (Figure 6H), which is in concordance with the results of DNA damage and H3K9me3 foci in Figure 6F and 6G. Moreover, the experimental result of A549 cells also proved that this SAHA treatment increased the radiosensitivities of both DCs-γ and DCs-α and their parent control, and it eliminated radioresistance of DCs-γ as well (Figure 7E). Therefore, it can be deduced that a higher steady state of HDAC activity together with well conserved histones and condensation chromatin structure in nuclear could be the prominent determinants of cell radioresistance.

## DISCUSSION

A number of previous studies have implicated the function of compact chromatin architecture in promoting cellular radioresistance [16, 17]. However, there is no literature concerning the role of radiation-induced chromatin remodeling in regulating radiosensitivity of DCs after low- and high-LET irradiation. This study provided new evidence that the chromatin remodeling is associated with radioresistance of DCs derived from irradiated cells.

In comparison with low-LET irradiation, a high-LET irradiation is able to elicit same effect at much lower doses and hence is more destructive to cells. Considering this LET-dependent effect, two lung cell lines were irradiated with equivalent doses of g-rays and a-particles to generate their DCs for radiosensitivity investigation. The measurement results of survival, cell proliferation and the formation of DNA damage-related γH2AX foci demonstrated that DCs-γ are more radioresistant than DCs-α. In general, the enhancement of radioresistance might be a result of a clonal selection process, including a better efficiency of DNA-damage repair systems [18], a higher level of anti-apoptotic [19], pro-survival factors [20], and especially enrichment of cancer stem cells (CSCs) [21–24]. However, our results showed that there was no significant alteration of the radiosensitivity of DCs-α in comparison with its parent cells without priming irradiation. This could result from the special biological characteristics of high-LET radiation such as less variation in cell cycle-related radiosensitivity and less repair capacity of radiation damage [25]. Therefore, there may be no clonal selection process in cells irradiated by high-LET rays. The findings of high-LET radiation superior to low-LET radiation in avoiding radioresistance of DCs may illustrate the advantages of using high-LET particles in tumor radiotherapy.

We found that the radioresistance of DCs-γ may be linked with chromatin remodeling after low-LET irradiation. Some other studies have indicated that the chromatin structure has significant influence on cell radiosensitivity. The chromatin has a compacted patch structure in radioresistant cells while it shows a loose sheet or a latticed structure in radiosensitive cells [26, 27]. It was reported that cell radioresistance was correlated strongly with the number of heterochromatin domains; the number of γH2AX foci remaining in the radioresistant sub-colonies was lower than that in the parent cells after X-ray irradiation, indicating that the increase of heterochromatin domain may be an indicator of radioresistance [28]. This is consistent with our results that the DCs-γ have a high level of heterochromatin domain and thus are radioresistant. The results that the level of the heterochromatin marker H3K9me3 was significantly increased in DCs-γ indicate that γ-ray irradiation allows chromatin to be more condensed and hence induces heterochromatinization eventually. The different outcomes in chromatin remodeling between low-LET and high-LET irradiations have also been observed. For DCs-α, the heterochromatin domain and H3K9me3 expression had similar levels to those in their parent cells without irradiation. These novel findings raise the value of deeply investigating the relationship between radiosensitivity and chromatin structure.

It is now appreciated that chromatin structure has an integral role in DSB repair and radiation response [29, 30]. Chromatin decondensation could be observed at sites of DSBs and manifested for 15 min post-irradiation as the decreased intensity of chromatin labeling, increased histone H4 lysine 5 acetylation and decreased H3K9me3. But after 40 min post-irradiation, these alterations in histone modifications were exchanged as the decreased acetylation of histone H4 lysine 5 and increased expression of H3K9me3 [31]. Recent evidence has shown that, the compaction of chromatin protects DNA against radiation-induced DSB and hence the relaxation of chromatin structure is a possible reason for radiosensitization [32]. Our results in Figure 6 showed that, after two weeks of γ-ray irradiation but not α-particle irradiation, DSB repair has almost completed and the chromatin structure in DCs-γ became more condensed than that in its parental cells.

Why was the heterochromatin domain increased in DCs-γ but not in DCs-α? Heterochromatin domain, an important form of chromatin remodeling, was mainly decreased by histone acetylation [33–35]. Histone acetylation, controlled by HATs and HDACs, relaxes nucleosome and chromatin structures and promote gene expression [36–38]. It has been known that HDACs play crucial roles in gene transcription and most likely in all eukaryotic biological processes by regulating chromatin conformation and thus are critical in maintaining a dynamic equilibrium of protein acetylation and also exert profound effects on other protein posttranslational modifications. Deacetylation of histones and non-histones may change chromatin conformation or inhibit the activities of transcription factors leading to an inhibition in gene expression [39]. Studies have shown that DNA in decondensed chromatin is nearly twice more vulnerable to radiation than that in normal chromatin [40]. As expected, for vital cellular regulators, the activities of HDACs are tightly controlled through a multitude of mechanisms, among which the recruitment into different co-repressor complexes and the activation of deacetylase activity by protein-protein interactions or by post-translational modifications are particularly relevant ones. HDACs’ biological functions are strictly dependent on their enzymatic activity [41]. Therefore, a reasonable explanation why the heterochromatin domain was increased in DCs-γ but not in DCs-α might be associated with differential influence of high- and low-LET irradiations on the activity of HDACs enzyme. Compared with DCs-α, a higher HDAC activity was detected in DCs-γ, and the high level of heterochromatin domain maker H3K9me3 corresponds very well with the radioresistance of DCs-γ. Therefore, the activity of HDAC may have a direct significant effect on the radiation sensitivity of DCs.

To further confirm the above opinion, it is important to evaluate the influence of HDACi on radiation response of the DCs. Our results show that when the cells were treated with SAHA, the activity of HDAC was inhibited but the activity of Ac-H3 was extensive increased; meanwhile, the radioresistance of DCs-γ was eliminated together with significant increase of γH2AX foci formation. Accordingly, SAHA relaxes chromatin structure by decreasing heterochromatin domains and results in cell radiosensitization. These findings are consistent with previous reports that HDACi has functions in increasing histone acetylation, relaxing chromatin structure and leading to reversible decondensation of chromatin regions [13, 42, 43]. In fact, radiation sensitization of tumor cells could be enhanced by many kinds of HDACi that alters chromatin conformation and/or decreases DNA repair capacity [44–46]. However, in the present study, although the DCs-γ of A549 was more resistant than DCs-α and its parent cells, the ability of radioresistance of DCs-γ of A549 was still lower than that of DCs-γ of Beas-2B cells. The possible reason may because that profound epigenetic alterations have taken place in various cancer cells [47]. Therefore, the identification of specific alteration in chromatin modulator in cancer cells offers the opportunity to develop new targeted therapy method.

In conclusion, this is the first report demonstrating that radiosensitivity can be reduced in DCs-γ but not DCs-α due to increases of heterochromatinization and HDAC activity. These findings support that the use of HDACi eliminates the radioresistance of DCs-γ by increasing histone acetylation, relaxing chromatin structure and leading to reversible decondensation of chromatin. Although a high-LET irradiation is superior to low-LET radiation in killing tumor cells, the combination of ionizing radiation with targeted HDACi treatment may become a potential effective cancer therapy strategy, especially for low-LET photon fraction radiotherapy.

## MATERIALS AND METHODS

### Cell culture

Human bronchial epithelial cell line Beas-2B cells were obtained as a gift from Nanjing Medical University. Human non-small cell lung cancer cell line A549 was purchased from Shanghai Cell Bank (Shanghai, China). Beas-2B cells were cultured in DMEM (HyClone, Beijing, China) supplemented with penicillin/streptomycin and 10% fetal bovine serum (FBS, Gibco Invitrogen, Grand Island, NY, USA). A549 cells were cultured in RPMI 1640 medium (HyClone, Beijing, China) supplemented with serum and antibiotics as described above. All the cell lines were grown at 37°C in the presence of 5% CO2 in a humidified incubator.

### Cell irradiation and establishment of DCs

Exponentially growing cells were exposed to γ-rays and α particles, respectively. Gamma-rays (0.23keV/μm) were generated from a ^137^Cs irradiator (Gammacell-40, Nordion International Inc., Kanata, Ontario, Canada) with a dose rate of 0.75Gy/min [48]. A ^241^Am α-particle plate source (Atom High Tech Co., Ltd., Beijing, China) was applied for α-irradiation. Because of the limited range of α-particles, cells were seeded on a Mylar-film based dish that was pre-coated with 150–300 kD poly-lysine (Sigma-Aldrich) and maintained overnight to allow cell attachment. After penetrating through three layers of 2.5μm thickness Mylar-film (DuPont, Wilmington, DE, USA), the energy of α-particle was 4.4 MeV with a LET of 100 keV/μm at the cell layer and the dose rate of α-irradiation was 0.244 Gy/min [49]. Cells were irradiated with 1 Gy α-particles or 6 Gy γ-rays. Because many cells were killed after 1 Gy of α-particles or 6 Gy of γ-rays irradiation, to obtain stable daughter cells (DCs), the irradiated cells were passaged every 3 days and cultured for 2 or 3 weeks until no suspension of dead cells in medium. Then, these survived cells after 2 or 3 weeks of priming irradiation of 1 Gy α-particles and 6 Gy γ-rays were defined as DCs-α and DCs-γ, respectively.

### Drug treatment

Beas-2B and A549 cells were treated with suberoylanilide hydroxamic acid (SAHA, Tocris Bioscience, Bristol, Avon, UK) of different concentrations from 0 to 10 μM for different time from 12 h to 72 h. SAHA is an inhibitor of HDAC enzyme. As a solvent of SAHA, 0.1% DMSO was used as a negative control (NC) of SAHA. After the pilot experiments for choosing a suitable drug treatment condition, Beas-2B cells or A549 cells were treated with 2 μM SAHA for 12 h before irradiation. After irradiation, the cells were washed with PBS triply and resuspended in fresh culture medium for further analysis.

### Cell proliferation assay

Cell proliferation was determined with Cell Counting Assay Kit-8 (CCK-8, Dojindo, Kumamoto, Japan). After each treatment, Beas-2B cells were seeded into 96-well microplate at 3×10^3^ cells/well and further cultured for 4 days at 37°C with 5% CO_2_. Then, 10 μl of kit reagent was added to each well followed by incubation for 2 h at 37°C. Cell proliferation was assessed by measuring the optical density (OD) at 450 nm of the culture medium with a plate reader (Infinite M200Pro, Tecan, Mannedorf, Switzerland). All results were normalized to the OD value of identical plain medium. Relative cell proliferation rate was defined as (OD value of the treated cells-OD value of the background control wells) / (OD value of the control cells-OD value of the background control wells). This CCK-8 assay was repeated triply and each trial was performed in six wells.

### Colony formation assay

Cell survival was measured by using colony formation assay. After irradiation, identified number of cells were re-seeded into 6-well plate and incubated at 37°C for 2 weeks to form colonies. The colonies were fixed with methanol for 20–30 min and stained with 0.1% crystal violet for 30 min in order to count. The cell survival fraction was calculated as the ratio of the plating efficiency (PE) of irradiated cells to the PE of control without irradiation.

### Western blotting assay

The total proteins of both cell lines were extracted and measured by Western blot analysis with the following antibodies: anti-H3K9me3 (Abcam, MA, USA; 1:1000), anti-HP1α (Abcam, MA, USA; 1:2000), anti-Ac-H3 (CST, 1:1000) and anti-β-Tubulin (Beyotime Biotechnology, 1:1000). The cells were washed triply with ice-cold PBS and treated with RIPA lysis buffer with 100 mM phenylmethanesulfonyl fluoride (PMSF) (Beyotime). An equal amount of total protein was subjected to 12% sodium dodecyl sulfate polyacrylamide gel electrophoresis (SDS-PAGE) and transferred to a polyvinylidene fluoride (PVDF) membrane (Millipore, Bedford, MA, USA) then probed with primary antibodies overnight at 4°C. The membranes were then incubated for 1 h with the secondary HRP-conjugated antibodies (1:1000; Cell Signaling Technology). β-Tubulin was used for the loading control. The protein bands were visualized using the ChemiDoc XRS system (Bio-Rad Laboratories, Hercules, CA, USA) and their densities were measured using the Quantity One software (Bio-Rad Laboratories).

### Immunofluorescence assay

Exponentially growing cells on coverslips were washed with PBS triply followed by fixation with 4% paraformaldehyde at room temperature for 15 min. Cells were permeabilized with 0.5% Triton X-100 solution for 15 min at room temperature and then blocked with 10% normal goat serum for 1 h. Primary antibodies with appropriate dilutions (H3K9me3, 1:500; γH2AX, 1:500) were added and incubated at 4°C overnight. Cells were then washed with PBS and probed with FITC/PI-conjugated secondary antibodies (1:1000) at room temperature for 1 h. Cell nuclear were counterstained with DAPI for 5 min and examined using a Zeiss Axioplan fluorescence microscope.

### Fluorometric assay of HDAC activity

Cell nucleus protein was extracted in a native lysis buffer for HDAC activity assay using a HDAC Activity Fluorometric Assay Kit (BioVision, USA) according to the manufacturer's instruction [50]. In some experiments, cells were incubated with SAHA of different concentrations for 12 h before nuclear protein extraction. The mean fluorescence of three independent experiments (each conducted in triplicate) was used to determine HDAC activity. The OD value of control without any treatment was measured together with the irradiated cells at each time point after irradiation. The relative activity of HDAC at each time points was defined as (OD value of the irradiated cells - OD value of the background control) / (OD value of the control cells - OD value of the background control).

### Statistical analysis

All data were obtained from 3 to 5 independent experiments and were presented as means ± S.E. Student's t-tests are used to perform statistical analysis using SPSS 17.0 software. *P* < 0.05 was considered as significant difference between indicated groups.
